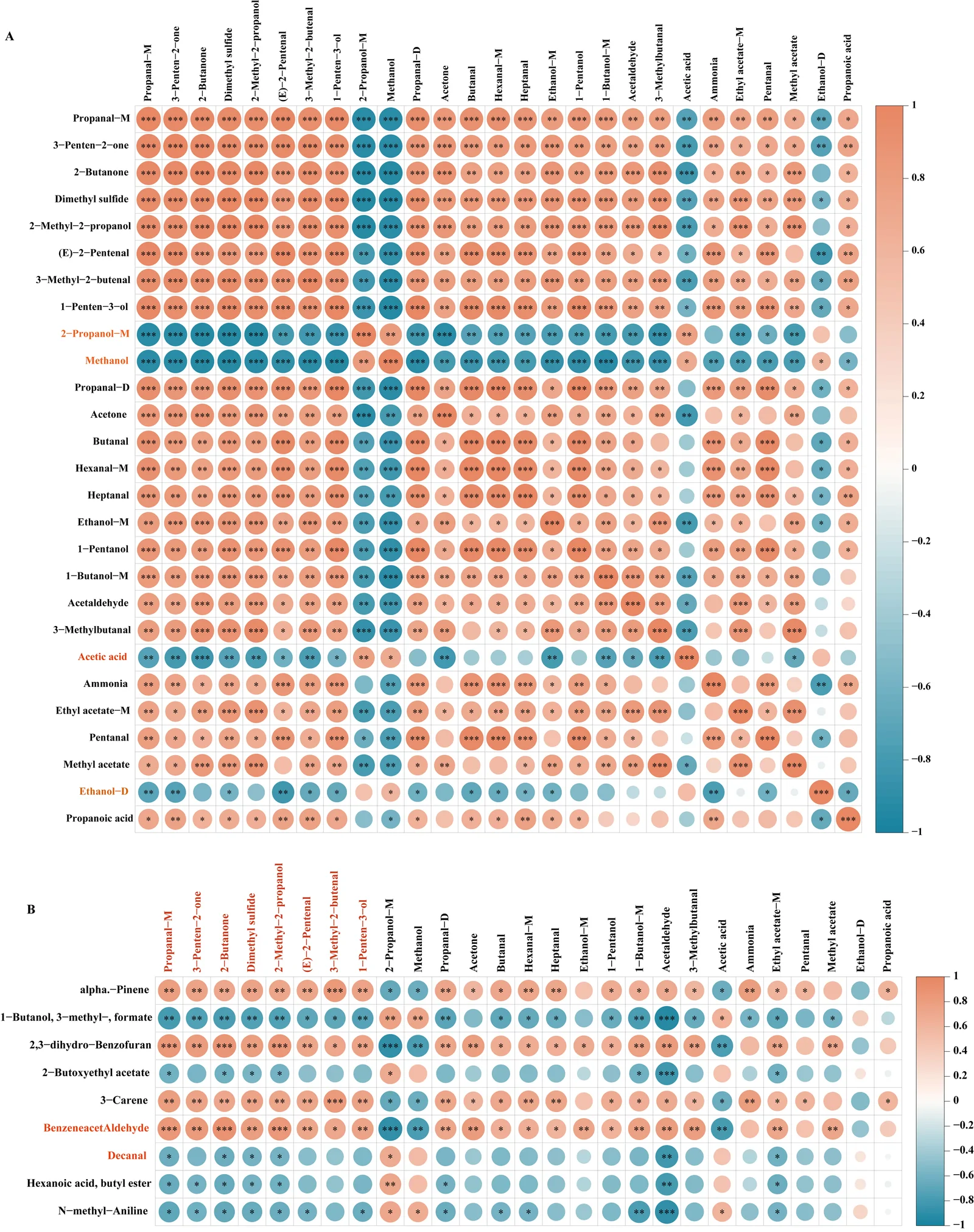# Lipid remodeling is associated with aroma formation during thermal processing of quail egg yolk: an integrated multi-omics study

**DOI:** 10.1016/j.crfs.2026.101559

**Published:** 2026-09-12

**Authors:** Cui Ma, Xiaoyue Yu, Jinsong Pi, Hao Zhang, Yuejin Pu, Wentao Ke, Yan Wu

**Affiliations:** a Institute of Animal Husbandry and Veterinary Medicine, Hubei Academy of Agricultural Sciences, Wuhan, 430064, China; b Key Laboratory of Animal Embryo Engineering and Molecular Breeding of Hubei Province, Wuhan, 430064, China; c Hubei Provincial Animal Husbandry Technology Promotion Station, Wuhan, 430084, China

**Keywords:** Quail egg yolk, Thermal processing, Glycerophospholipid remodeling, Volatile compounds, Multi-omics

## Abstract

Thermal processing substantially influences egg flavor quality, yet the metabolic basis underlying aroma development in quail egg yolk remains incompletely understood. In this study, GC–IMS, HS-SPME–GC–MS, lipidomics, and widely targeted metabolomics were integrated to characterize flavor-related metabolic changes during thermal processing. GC–IMS detected 48 VOCs, whereas HS-SPME–GC–MS detected 451 volatile features, of which 161 compounds with available odor threshold data were further evaluated by OAV analysis. Aldehydes, ketones, esters, and terpenoids predominated in cooked yolks. GC–IMS characterized the volatile fingerprint associated with thermal processing, whereas HS-SPME–GC–MS identified benzeneacetaldehyde and decanal as candidate odor-active compounds. Lipidomic analysis revealed extensive remodeling of glycerophospholipids and glycerolipids during heating, accompanied by marked alterations in amino acids, organic acids, and sugar derivatives. Differential metabolites annotated to fatty acid metabolism, α-linolenic acid metabolism, and pentose and glucuronate interconversions were associated with changes in volatile profiles. Multi-omics integration further revealed coordinated associations among lipid remodeling, changes in water-soluble metabolites, and aroma-associated VOCs. In particular, changes in glycerophospholipid species were closely correlated with aldehydes and other characteristic volatiles, highlighting a potential association between phospholipid remodeling and aroma development during thermal processing. Collectively, these findings provide an integrated metabolic perspective on aroma formation in cooked quail egg yolk and provide a basis for understanding the associations between lipid remodeling and volatile evolution during thermal processing.

## Highlights

• Thermal processing extensively remodels membrane glycerophospholipids in quail egg yolk.
• Lipid remodeling is associated with coordinated changes in flavor-related metabolites.
• Lipid oxidation and Maillard-related reactions are associated with cooked aroma development.
• Integrated multi-omics reveals coordinated associations among lipids, metabolites, and aroma compounds.

## Graphical abstract

**Figure:**
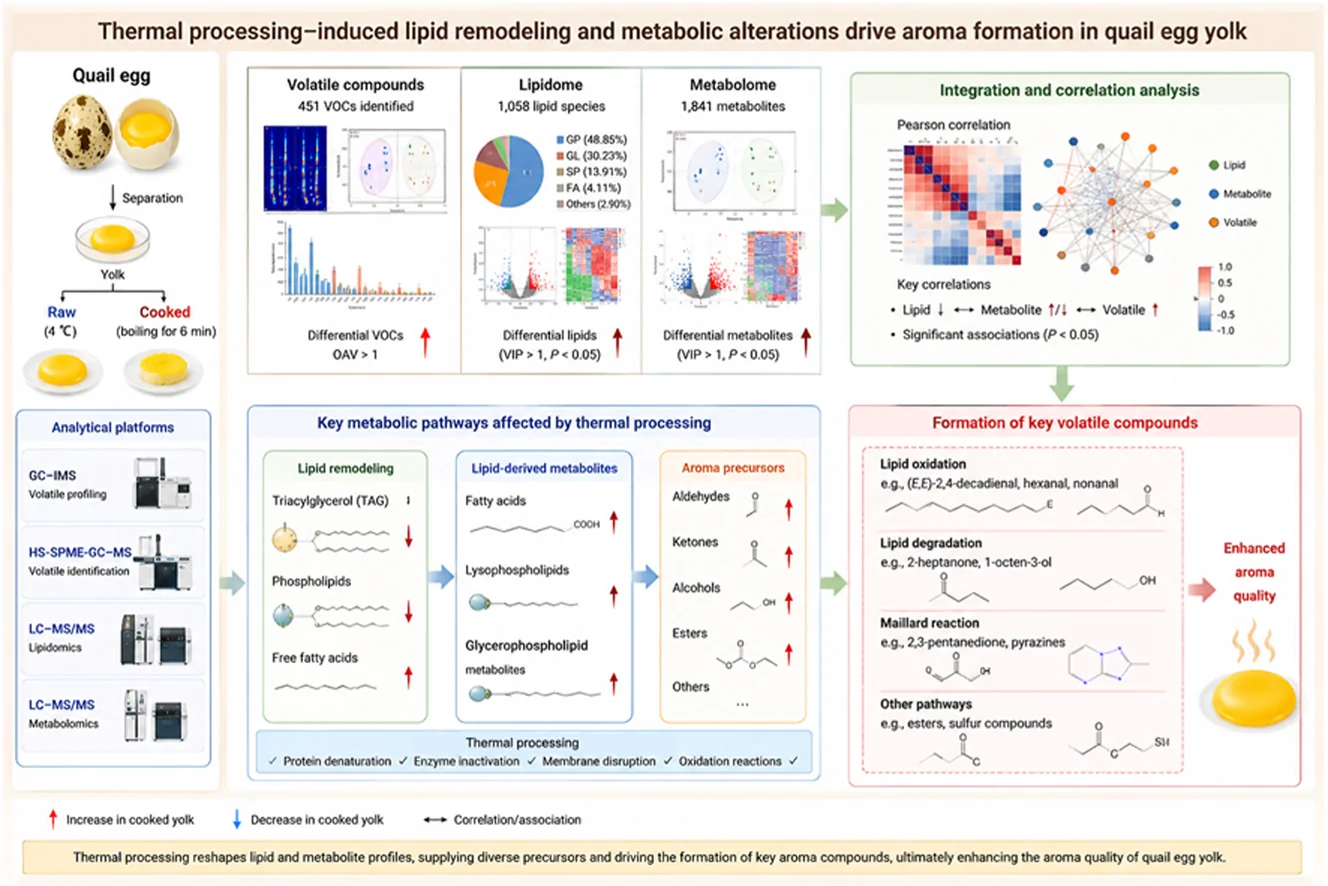

## 1 Introduction

Quail eggs are a nutrient-dense food rich in lipids, proteins, and bioactive compounds, with the yolk representing an important reservoir of flavor precursors (Czerwonka et al., 2024). Flavor is a key determinant of consumer acceptance and product quality, but it is strongly influenced by thermal processing. During cooking, yolk components undergo complex chemical transformations, including lipid degradation, Maillard reactions, and interactions among amino acids, sugars, and other metabolites, leading to changes in volatile composition (Zhou et al., 2025). However, the mechanisms responsible for heat-induced flavor development in quail egg yolk remain poorly understood. In particular, the relationships among lipid transformation, metabolite alterations, and volatile generation during thermal processing require further investigation.

Lipid oxidation and Maillard reactions are widely recognized as important chemical processes associated with flavor development during thermal processing. Lipid oxidation generates aldehydes, ketones, alcohols, and other volatile compounds, such as hexanal and nonanal, which contribute to cooked aroma characteristics (Li et al., 2025; Ping et al., 2018). Meanwhile, reactions between amino acids and reducing sugars produce pyrazines, furans, and other thermally derived flavor compounds (Ping et al., 2018; Zhang et al., 2024). These processes are affected by the lipid composition and metabolic status of the yolk and may jointly influence aroma formation (Li et al., 2025). However, conventional approaches based mainly on gas chromatography-mass spectrometry (GC–MS) or gas chromatography-ion mobility spectrometry (GC–IMS) provide limited information on the metabolic changes associated with volatile formation. Therefore, an integrated analysis of volatiles, lipids, and metabolites is needed to better understand heat-induced flavor development in egg yolk.

Recent advances in analytical technologies have provided new opportunities to investigate flavor formation from both chemical and metabolic perspectives (Kubo et al., 2023). GC–IMS enables rapid fingerprinting of volatile profiles and discrimination of aroma differences between raw and heated yolk samples (Song et al., 2025a, Song et al., 2025b), while HS-SPME-GC–MS allows accurate identification of individual volatile compounds and their odor contributions (Zhang et al., 2018). However, volatile analysis alone cannot fully explain the metabolic origins of aroma compounds. Lipidomics and metabolomics provide complementary information by characterizing lipid and metabolite alterations potentially associated with flavor development. Despite increasing interest in egg flavor research, the relationship between lipid remodeling, metabolite changes, and volatile generation during thermal processing of quail yolk remains unclear. Therefore, this study integrated GC–IMS, HS-SPME-GC–MS, lipidomics, and widely targeted metabolomics to characterize thermally induced flavor changes in quail egg yolk. The volatile profiles, lipid alterations, and metabolite variations were analyzed, and correlation analyses were conducted to explore the associations among lipid species, metabolites, and volatile compounds. This work provides insights into the associations between lipid remodeling and aroma formation during thermal processing and offers a multi-omics perspective on flavor development in cooked quail egg yolk. Because the present study was conducted under a single standardized boiling condition, the resulting findings should be interpreted within the context of this specific thermal treatment.

## 2 Materials and methods

### 2.1 Sample preparation and chemical reagents

Fresh quail eggs (12.0-15.0 g) were obtained from Shendan Company (Hubei, China) within 24 h of laying and stored at 4 °C before processing. For the cooked-yolk group, intact whole quail eggs were immersed in distilled water at 15 °C (egg-to-water ratio, 1 egg:40 mL) and heated from cold water using an induction cooker (maximum power, 2400 W). Heating time was recorded from the onset of continuous boiling, and the eggs were maintained under continuous boiling for 6 min. Immediately after heating, the eggs were transferred to an ice-water bath to rapidly terminate thermal processing. After cooling to room temperature, the yolks were carefully separated from the albumen, homogenized individually, aliquoted into sterile cryogenic tubes, snap-frozen in liquid nitrogen, and stored at −80 °C until analysis. Each treatment consisted of six independent biological replicates (n = 6), with each biological replicate obtained from a separate quail egg, unless otherwise specified.

Analytical-grade n-hexane, NaCl, and formic acid were used for sample preparation. Chromatography-grade solvents, including methanol, acetonitrile, isopropanol, chloroform, and methyl tert-butyl ether (MTBE), were employed. Lipid standards and n-alkanones (2-butanone, 2-pentanone, 2-hexanone, 2-heptanone, 2-octanone, 2-nonanone; all analytical grade) were purchased from Aladdin (Shanghai, China). High-purity nitrogen gas (≥99.999%) was used as the carrier and drift gas for GC–IMS. Headspace vials (20 mL) were sourced from Haineng Scientific (Shandong, China).

Volatile compounds were analyzed using a FlavourSpec® GC–IMS system (G.A.S., Dortmund, Germany) equipped with a CTC-PAL 3 static headspace autosampler (CTC Analytics AG, Zwingen, Switzerland), with data acquired and processed using VOCal software (v0.4.03; G.A.S.). GC–MS/MS analysis was performed on an Agilent 8890–7000E system with a DB-5MS capillary column, using SPME fiber extraction and automated sample handling. Lipidomic and widely targeted metabolomic analyses were conducted on a QTRAP 6500+ LC–MS/MS platform (SCIEX), supported by a refrigerated centrifuge, vacuum concentrator, vortex mixer, ultrasonic cleaner, and other standard laboratory equipment.

### 2.2 GC–IMS analysis

Volatile compounds in quail egg yolk samples were analyzed using a gas chromatography–ion mobility spectrometry system (GC–IMS, FlavourSpec®, G.A.S., Dortmund, Germany) following previously reported procedures with minor modifications (Wang et al., 2020; Zhang et al., 2025). For each treatment, six independent biological replicates (n = 6) were analyzed. For analysis, 2.0 g of sample was accurately weighed into a 20 mL headspace vial and sealed immediately. The samples were incubated at 60 °C for 20min with continuous agitation at 500 rpm to allow volatile compounds to equilibrate in the headspace.

Following incubation, 500 μL of the headspace gas was automatically injected into the GC–IMS system in splitless mode using a heated syringe at 85 °C. Chromatographic separation was carried out on a capillary column maintained at a constant temperature of 60 °C, with the injector temperature set at 80 °C. High-purity nitrogen (≥99.999%) was used as the carrier gas, and the flow rate was programmed as follows: 2.0 mL/min for 2 min, increased linearly to 10.0 mL/min over 8min, then ramped to 100.0 mL/min over 10 min, and finally maintained at this level to complete a total run time of 50 min. The ion mobility spectrometry was operated in positive ion mode with a tritium (^3^H) ionization source. The drift tube (length: 53 mm) was maintained at 45 °C under an electric field strength of 500 V/cm. Nitrogen (≥99.999%) was used as the drift gas at a constant flow rate of 75 mL/min. A homologous series of n-ketones (C4–C9) was used for retention index (RI) calibration, and volatile compounds were tentatively identified by comparing RI and drift time with the built-in GC–IMS database (NIST 2020 and IMS library) using VOCal software (G.A.S., Germany).

### 2.3 HS-SPME-GC-MS analysis

Volatile compounds in quail egg yolk were analyzed using headspace solid-phase microextraction coupled with gas chromatography–mass spectrometry (HS-SPME–GC–MS) following previously reported procedures with minor modifications (Jia et al., 2025). Prior to extraction, samples were cryogenically ground in liquid nitrogen to obtain a homogeneous powder. Approximately 0.2 g of sample was transferred into a 20 mL headspace vial (Agilent, Palo Alto, CA, USA), followed by the addition of 0.2 g NaCl to enhance volatile release and inhibit enzymatic activity. The vial was immediately sealed with a TFE–silicone septum. The samples were equilibrated at 60 °C for 5 min, after which a DVB/CWR/PDMS fiber (120 μm, Agilent, USA) was exposed to the headspace for 15 min at the same temperature. Following extraction, the fiber was inserted into the GC injector and thermally desorbed at 250 °C for 5 min in splitless mode.

GC–MS analysis was carried out using an Agilent 8890 gas chromatograph coupled with a 7000E triple quadrupole mass spectrometer (Agilent Technologies, USA). Separation was achieved on a DB-5MS capillary column (30 m × 0.25 mm × 0.25 μm). High-purity helium (≥99.999%) was used as the carrier gas at a constant flow rate of 1.2 mL/min. The oven temperature program was set as follows: initial temperature at 40 °C (held for 3.5 min), increased to 100 °C at 10 °C/min, then to 180 °C at 7 °C/min, and finally ramped to 280 °C at 25 °C/min and held for 5 min. The injector, ion source, quadrupole, and transfer line temperatures were maintained at 250 °C, 230 °C, 150 °C, and 280 °C, respectively. Mass spectrometric detection was performed under electron impact (EI) ionization at 70 eV in selected ion monitoring (SIM) mode.

Each compound was confirmed based on retention time matching and characteristic ion fragments (one quantifier ion and two to three qualifier ions), following established volatilomics identification criteria (David and Sparkman, 2005; Schymanski et al., 2014). Semi-quantitative analysis was conducted using an isotopically labeled internal standard (benzaldehyde-d_6_). The concentration of each compound was calculated based on the ratio of its peak area to that of the internal standard using the following equation:

$$X_{i}=\frac{V_{s}\times C_{s}}{M}\times \frac{I_{i}}{I_{s}}$$

where $X_{i}$ represents the concentration of compound i (μg/g), $V_{s}$ and $C_{s}$ denote the volume and concentration of the internal standard, respectively, M is the sample mass, and $I_{i}$ and $I_{s}$ are the peak areas of the analyte and internal standard.

Volatile compounds potentially associated with aroma differences were evaluated based on their semi-quantitative abundance, VIP values, statistical significance, fold changes, and reported odor characteristics. For compounds with available odor thresholds reported in the literature, odor activity values (OAVs) were additionally estimated following previously reported studies (Huang et al., 2022):

$$OAV_{i}=\frac{C_{i}}{T_{i}}$$

where Ci represents the semi-quantitatively estimated concentration and Ti represents the odor threshold reported in the literature.

Because compound concentrations were estimated by semi-quantification rather than authentic calibration, and odor thresholds were obtained from the literature rather than determined in the egg yolk matrix, OAV values were regarded only as supportive information for discussing potential aroma contributors. They were not interpreted as absolute measures of aroma contribution or used as the sole criterion for identifying key aroma compounds.

### 2.4 Targeted lipidomic analysis

Frozen samples were thawed on ice, and targeted lipidomic analysis was performed following previously reported procedures with minor modifications (Cajka and Fiehn, 2014; Matyash et al., 2008). Approximately 20 mg of tissue was extracted with methyl tert-butyl ether/methanol (3:1, v/v) containing internal standards. After vortexing, water was added to induce phase separation, followed by centrifugation (4000 × g, 10min). The upper organic layer was collected, evaporated to dryness, and reconstituted in acetonitrile/isopropanol (1:1, v/v) for LC–MS/MS analysis.

Lipid separation was performed on an Accucore C30 column (2.6 μm, 2.1 × 100 mm; Thermo Fisher Scientific) using an ExionLC AD UPLC system coupled to a QTRAP 6500+ mass spectrometer (SCIEX). Mobile phases were acetonitrile/water (60:40) and acetonitrile/isopropanol (10:90), both containing 0.1% formic acid and 10 mM ammonium formate, applied with a gradient elution. The flow rate was 0.35 mL/min, column temperature 45 °C, and injection volume 2 μL. Mass spectrometry was operated in both positive and negative ESI modes under MRM acquisition, with source temperature 500 °C, spray voltage ±5500/−4500V, and optimized declustering potential and collision energy for each transition. Lipid species were annotated using a self-built database (MWDB; MetWare) with authentic standards, and quantification was performed in MRM mode using internal standards for signal normalization following established targeted lipidomics workflows (Thévenot et al., 2015).

### 2.5 Widely-targeted-metabolomics analyses

Frozen samples were thawed on ice, and approximately 20 mg of tissue was extracted with 400 μL of precooled methanol/water (7:3, v/v) containing internal standards. After vortexing and ultrasonic extraction in an ice bath, proteins were precipitated at −20 °C. Samples were then centrifuged (12,000 rpm, 4 °C), and the supernatant was clarified by a second centrifugation before being aliquoted for LC–MS/MS analysis (Chen et al., 2013).

Metabolites were separated on a Waters ACQUITY HSS T3 C18 column (1.8 μm, 2.1 × 100 mm) using an ExionLC AD UPLC system coupled to a QTRAP LC–MS/MS (SCIEX). Mobile phases consisted of 0.1% formic acid in water (A) and acetonitrile (B), with a linear gradient from 5% to 99% B. The flow rate was 0.4 mL/min, column temperature 40 °C, and injection volume 2 μL. Mass spectrometry was performed in both positive and negative ESI modes using MRM acquisition, with source temperature 500 °C, ion spray voltage ±5500/−4500 V, and optimized declustering potentials and collision energies for each transition.

### 2.6 Statistical analysis

All experimental data are presented as mean ± standard error (SE) based on six independent biological replicates (n = 6). Statistical analyses were performed using Matlab (MathWorks, Natick, MA, USA). Differences between raw and cooked yolk samples were evaluated using independent-sample t-tests, and *P* < 0.05 was considered statistically significant. GC–IMS data were processed using the VOCal software package (G.A.S., Germany), including the Reporter, Gallery Plot, and Dynamic PCA/PLS-DA modules, to generate three-dimensional spectra, topographic plots, difference spectra, fingerprint profiles, and multivariate analyses for comparative evaluation of volatile profiles. Support vector machine (SVM) analysis was performed using the Wekemo Bioincloud platform (Gao et al., 2024) solely as a feature selection approach to rank candidate discriminant volatile compounds according to their relative importance, rather than to construct or validate a predictive classification model. Feature importance was evaluated based on the average importance scores generated by the SVM algorithm. Accordingly, no predictive performance metrics (e.g., classification accuracy, sensitivity, specificity, or uncertainty) were evaluated in the present study. For lipidomic and metabolomic analyses, principal component analysis (PCA) and partial least squares-discriminant analysis (PLS-DA) were performed using MetaboAnalyst. Differential lipid species and metabolites were identified based on VIP > 1 combined with *P* < 0.05. Pearson correlation analysis was performed to evaluate associations among differential volatile compounds, lipid species, and metabolites. Correlation heatmaps and network visualizations were generated using R software (v4.4.3). All supplementary tables were independently cross-checked by two co-authors against the corresponding original datasets and statistical outputs to verify the accuracy of group assignments, table headers and labels, numerical values, and statistical information before finalization.

## 3 Results and discussion

### 3.1 GC–IMS reveals global volatile remodeling during thermal processing of quail egg yolk

**Fig. 1:**
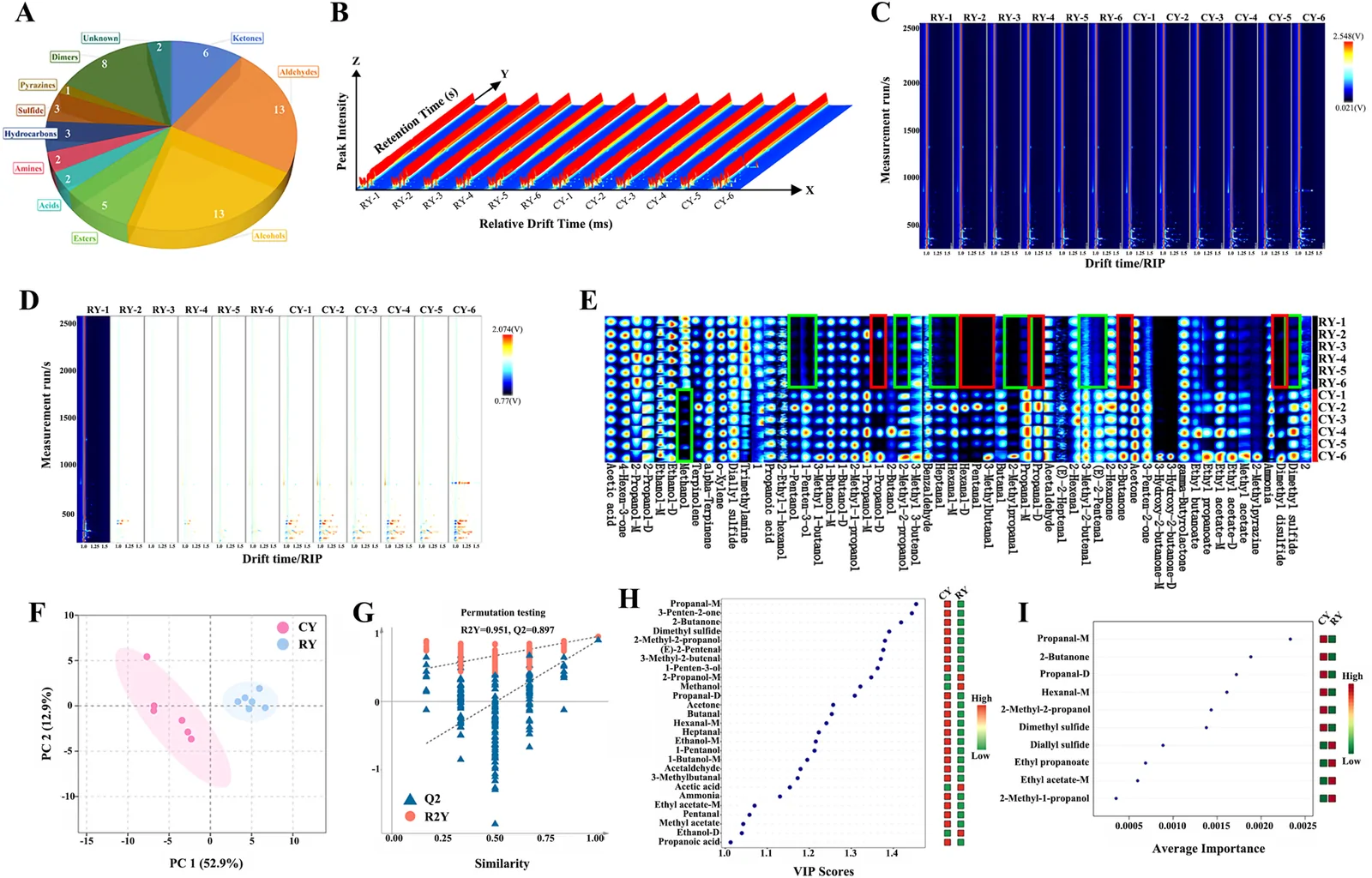
**GC–IMS analysis of volatile organic compounds (VOCs) in raw (RY) and cooked (CY) quail egg yolk.** (A) Classification and relative proportions of identified VOCs in raw and cooked yolks. (B) Three-dimensional topographic plots showing VOC distributions based on retention time, drift time, and signal intensity. (C) Two-dimensional peak plots illustrating VOC distribution patterns along retention and drift time axes. (D) Comparison of normalized signal intensities of representative VOCs (e.g., 2-butanone and ethanol) between raw and cooked yolks. (E) GC–IMS fingerprint profiles of yolk samples. Red rectangles indicate VOCs detected exclusively in one sample group, whereas green rectangles indicate VOCs exhibiting pronounced differences in signal intensity between the two sample groups. (F) Partial least squares discriminant analysis (PLS-DA) score plot showing separation of VOC profiles between raw and cooked yolks. (G) Model performance evaluation based on accuracy, R^2^, and Q^2^ values across different numbers of components. (H) Variable importance in projection (VIP) scores of VOCs contributing to discrimination between raw and cooked yolks. (I) Support vector machine (SVM)-based feature ranking of VOCs for discrimination between RY and CY samples. The y-axis represents the top 10 VOC features ranked by importance, and the x-axis indicates the corresponding importance scores.

The volatile profiles of raw (RY) and cooked (CY) quail egg yolks were characterized using gas chromatography–ion mobility spectrometry (GC–IMS) (Song et al., 2025). A total of 58 VOCs were detected, of which 48 were identified, including aldehydes, alcohols, ketones, esters, and sulfur-containing compounds (Fig. 1A). Three-dimensional topographic plots and two-dimensional projections showed distinct differences in VOC distributions between RY and CY (Fig. 1B–D). In the 2D spectra, each signal represented an individual volatile compound, with color intensity reflecting relative abundance. Multiple signals corresponding to the same compound were mainly attributed to monomer and dimer forms caused by differences in proton affinity and drift behavior. Difference spectra further showed increased signal intensity and greater peak diversity in CY compared with RY, indicating substantial changes in volatile composition during thermal processing (Fig. 1E). Fingerprint analysis revealed six VOCs that were detected only in cooked yolks, including propanal (dimer), hexanal (dimer), pentanal, 3-methylbutanal, 2-butanone, and dimethyl disulfide. PLS-DA showed clear discrimination between RY and CY, with PC1 and PC2 explaining 65.8% of the total variance. Permutation tests confirmed the reliability of the model (R^2^ and Q^2^ values close to 1; Fig. 1F–G). Based on VIP values (>1), 27 VOCs were identified as major contributors to group separation (Fig. 1H). Aldehydes, ketones, esters, and sulfur-containing compounds, such as propanal-M, 3-penten-2-one, 2-butanone, dimethyl sulfide, (E)-2-pentenal, and 1-penten-3-ol, were enriched in CY, whereas alcohols and organic acids, including 2-propanol-M, methanol, ethanol-D, and acetic acid, were more abundant in RY. These results indicate that thermal processing shifted the volatile profile of quail egg yolk from an alcohol- and acid-rich state toward a more complex aroma profile dominated by aldehydes, ketones, and sulfur-containing compounds.

Pearson correlation analysis of the 27 VIP-selected VOCs (Fig. S1A), which presents the pairwise correlations among the major VOCs contributing to group discrimination, revealed distinct association patterns between compounds enriched in RY and CY. Alcohols and organic acids enriched in RY showed strong positive correlations with each other but were negatively correlated with CY-enriched aldehydes, ketones, and sulfur-containing compounds. This pattern suggests coordinated remodeling of volatile compounds during thermal processing, accompanied by the accumulation of oxidation- and heat reaction-derived volatiles and the reduction of native alcohols and organic acids. Such changes are consistent with previous studies showing that lipid oxidation and Maillard-related reactions are important chemical processes associated with aroma development in heated egg products (Maire et al., 2013; Troise et al., 2020). Oxidative degradation of unsaturated fatty acids has been reported to generate aldehydes such as hexanal, heptanal, nonanal, and (E)-2-pentenal, whereas interactions between lipid oxidation and amino acid degradation may further contribute to Strecker aldehydes (e.g., 3-methylbutanal and phenylacetaldehyde) and sulfur-containing volatiles such as dimethyl sulfide (Chen et al., 2019; Maire et al., 2013). These observations provide a plausible explanation for the enrichment of aldehydes, ketones, and sulfur-containing compounds in cooked yolks.

Although GC–IMS revealed substantial remodeling of the volatile profile after thermal processing, the molecular basis underlying these changes could not be resolved solely from volatile data. Previous studies have suggested that heat-induced aroma formation in egg yolk is closely associated with lipid oxidation and amino acid-derived reactions, which may contribute to changes in volatile composition during thermal processing (Fu et al., 2022; Song et al., 2025a, Song et al., 2025b). To further identify candidate discriminative VOCs associated with the separation between RY and CY, support vector machine (SVM) analysis was performed to rank the ten most important volatile features contributing to group discrimination (Fig. 1I). Compounds including 2-butanone, propanal-d, hexanal-m, 2-methyl-2-propanol, and dimethyl sulfide showed consistent differences between groups, suggesting that they may represent candidate VOCs associated with heat-induced flavor changes. These SVM-ranked VOCs were also highlighted by the PLS-DA and Pearson correlation analyses, indicating that they consistently contributed to the discrimination between raw and cooked yolks across multiple analytical approaches. Because GC–IMS and HS-SPME–GC–MS offer complementary analytical strengths, GC–IMS was primarily used to characterize global volatile fingerprints and processing-related volatile changes, whereas HS-SPME–GC–MS served as the principal platform for semi-quantitative volatile profiling, identification of candidate volatile markers, and integration with lipidomic and metabolomic analyses. Overall, these GC–IMS results demonstrate that thermal processing induces global remodeling of the volatile profile of quail egg yolk, characterized by enrichment of aldehydes, ketones, and sulfur-containing compounds together with depletion of alcohols and organic acids.

### 3.2 HS-SPME–GC–MS identifies candidate aroma-active compounds associated with thermal processing

**Fig. 2:**
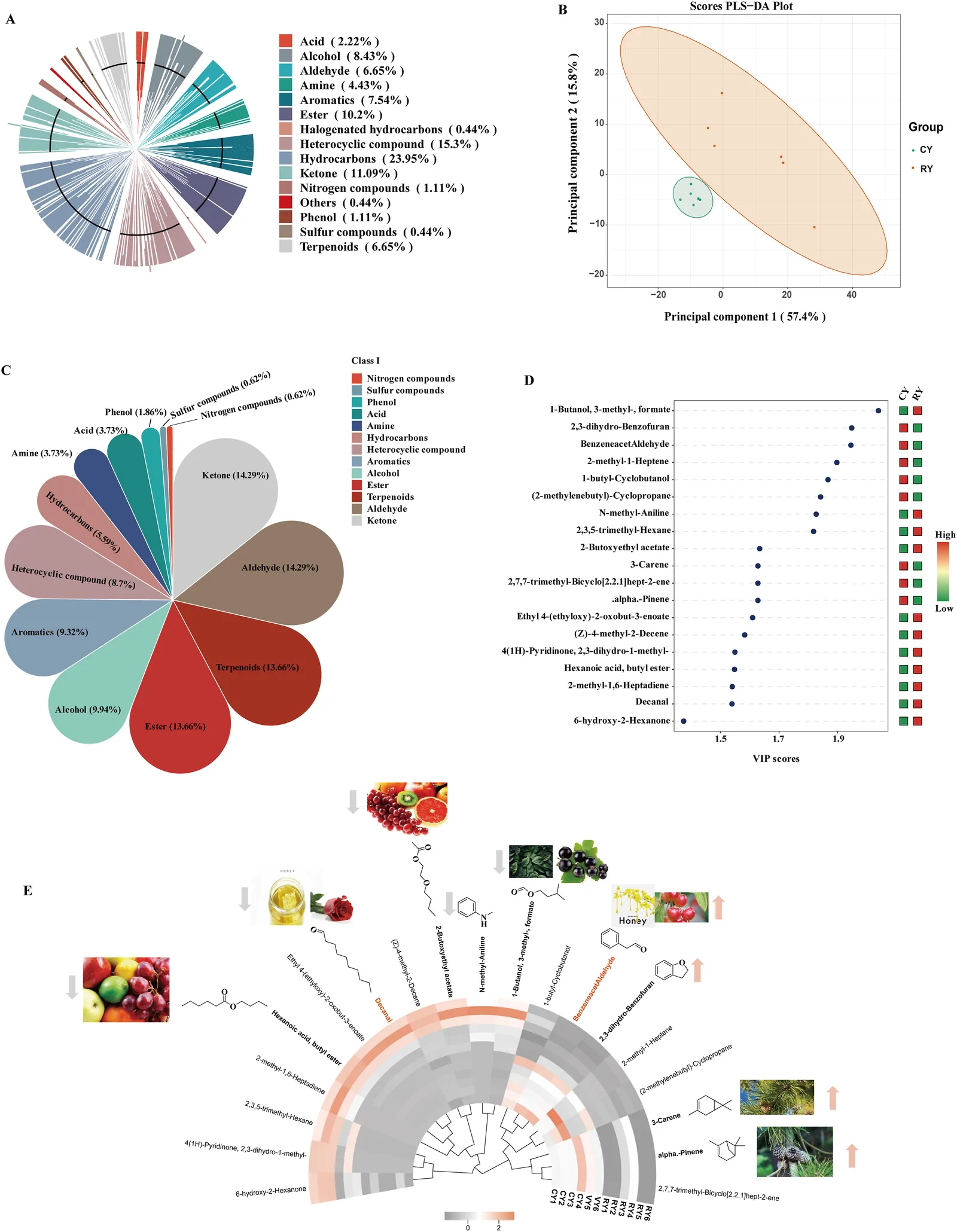
**Characterization of volatile compounds in raw (RY) and cooked (CY) quail egg yolks based on HS-SPME-GC–MS analysis.** (A) Classification and relative abundance of identified volatile compounds. (B) PLS-DA score plot showing separation of volatile profiles between RY and CY samples. (C) Circular plot showing the distribution of major volatile compound classes. (D) Variable importance in projection (VIP) score plot of the differential volatile organic compounds (VOCs) between the raw yolk (RY) and cooked yolk (CY) groups. VOCs are ranked according to their VIP scores, and the colored squares indicate the relative abundance of each compound in the two groups. (E) Odor wheel integrating selected volatile compounds with their reported aroma descriptors and observed abundance differences between the RY and CY groups. Upward and downward arrows indicate higher and lower relative abundances, respectively, in CY compared with RY and do not imply specific formation or degradation mechanisms.

HS-SPME–GC–MS was subsequently employed to further characterize the volatile composition of raw and cooked quail egg yolks. Volatile compounds were identified by matching mass spectra and retention indices with reference standards and the NIST library. After peak detection, deconvolution, and removal of redundant signals, 451 volatile features were detected (Fig. 2A). The complete list of identified volatile compounds is provided in Table S1, and the VIP-selected VOCs are presented in Table S2. These compounds were assigned to different chemical classes, including hydrocarbons (23.95%), heterocyclic compounds (15.3%), ketones (11.09%), esters (10.2%), alcohols (8.43%), aromatics (7.54%), aldehydes (6.65%), terpenoids (6.65%), amines (4.43%), and acids (2.22%), while nitrogen-containing compounds, phenols, sulfur compounds, halogenated hydrocarbons, and other minor components each accounted for less than 1%. The diverse distribution of VOC classes indicates the complexity of aroma composition in quail egg yolk. Many of these compounds have previously been associated with lipid oxidation, Maillard and Strecker reactions during thermal processing, although individual compounds may originate from multiple formation pathways (Li et al., 2025). PLS-DA showed clear discrimination between raw and cooked yolk samples (Fig. 2B), indicating that thermal processing substantially altered the volatile profiles (Jiang et al., 2025). These findings provide an overview of the volatile changes associated with thermal processing and a basis for subsequent comparison of aroma-related compounds.

To provide supplementary information on the potential sensory relevance of the detected VOCs, OAVs were estimated for compounds with available odor threshold data. Because matrix-specific odor threshold values for quail egg yolk are currently unavailable, odor thresholds were obtained from published literature and publicly available databases. These literature-derived thresholds may not accurately reflect odor perception in the egg yolk matrix because the high lipid and protein contents can influence volatile release and effective odor thresholds. Among the 451 volatile compounds detected by HS-SPME-GC–MS, OAVs were determined for 161 compounds with available odor threshold data, providing supplementary information regarding their potential sensory relevance (Fig. 2C). The complete list of volatile compounds with estimated OAVs is provided in Table S3. The compounds for which OAVs were estimated covered various chemical classes, including aldehydes (14.29%), ketones (14.29%), esters (13.66%), terpenoids (13.66%), alcohols (9.94%), aromatics (9.32%), heterocyclic compounds (8.70%), hydrocarbons (5.59%), amines (3.73%), acids (3.73%), phenols (1.86%), and nitrogen- and sulfur-containing compounds (0.62% each). Among the compounds for which OAVs were estimated, aldehydes and ketones accounted for the largest proportions (H. Li et al., 2025). This observation is consistent with previous studies reporting that lipid oxidation is an important source of volatile compounds during thermal processing. Esters and heterocyclic compounds, which contribute fruity and roasted notes, have frequently been reported in thermally processed foods and may be associated with multiple reaction pathways, including esterification as well as Maillard- and Strecker-related reactions, depending on the processing conditions (Lei et al., 2024; Shi et al., 2024), further enriching the aroma profile. To further identify VOCs associated with aroma differentiation between raw and cooked yolks, VIP analysis was performed. The top 30 VOCs with VIP values > 1 were selected as discriminant compounds between raw yolk (RY) and cooked yolk (CY) (Fig. 2D), highlighting major volatile markers associated with heat-induced flavor differentiation (Zhou et al., 2024a, Zhou et al., 2024b). Further analysis using VIP values (>1) together with *P* < 0.05 identified 19 significantly altered VOCs, nine of which had estimated OAV values greater than 1 (Fig. 2E). These VOCs are summarized in Table S4. It should be noted that the presence of individual volatile compounds in raw yolks does not necessarily indicate their formation through thermal reactions, because several compounds commonly associated with Maillard or Strecker chemistry may also arise from endogenous metabolism, lipid oxidation–amino acid interactions, or other non-thermal pathways. Among the significantly altered VOCs, α-pinene, 2,3-dihydrobenzofuran, 3-carene, and benzeneacetaldehyde were enriched in cooked yolks, whereas 1-butanol, 3-methyl-, formate, 2-butoxyethyl acetate, decanal, hexanoic acid butyl ester, and N-methylaniline were more abundant in raw yolks. It should also be noted that the volatile compounds reported by HS-SPME–GC–MS represent only those confidently identified under the present analytical conditions. Therefore, the absence of individual volatile compounds from the GC–MS results should not be interpreted as evidence that they were absent from the samples. Although the increased abundance of α-pinene and 3-carene remains difficult to explain, heating may alter the yolk matrix and the partitioning behavior of pre-existing volatile compounds, thereby increasing their release into the headspace during HS-SPME analysis. However, there is currently insufficient evidence to determine whether these compounds are newly formed during thermal processing or simply become more readily detectable after heating. Therefore, no further mechanistic interpretation is made in the present study. In contrast, benzeneacetaldehyde, a floral and honey-like volatile reported to arise predominantly from phenylalanine degradation during thermal processing (Wang et al., 2025), exhibited a marked increase after cooking, whereas decanal was more abundant in raw yolks. Considering their significant changes after cooking, estimated OAV values, and reported odor characteristics, benzeneacetaldehyde and decanal were regarded as representative volatile markers associated with aroma differentiation between raw and cooked quail egg yolks. Because the OAV values were estimated from semi-quantitative data using literature-derived odor thresholds rather than matrix-specific threshold values, they should be interpreted as relative estimates of the potential sensory relevance of volatile compounds. Accordingly, these compounds were regarded as candidate volatile markers rather than definitive key aroma compounds.

### 3.3 Lipidomic remodeling of quail egg yolk during thermal processing

**Fig. 3:**
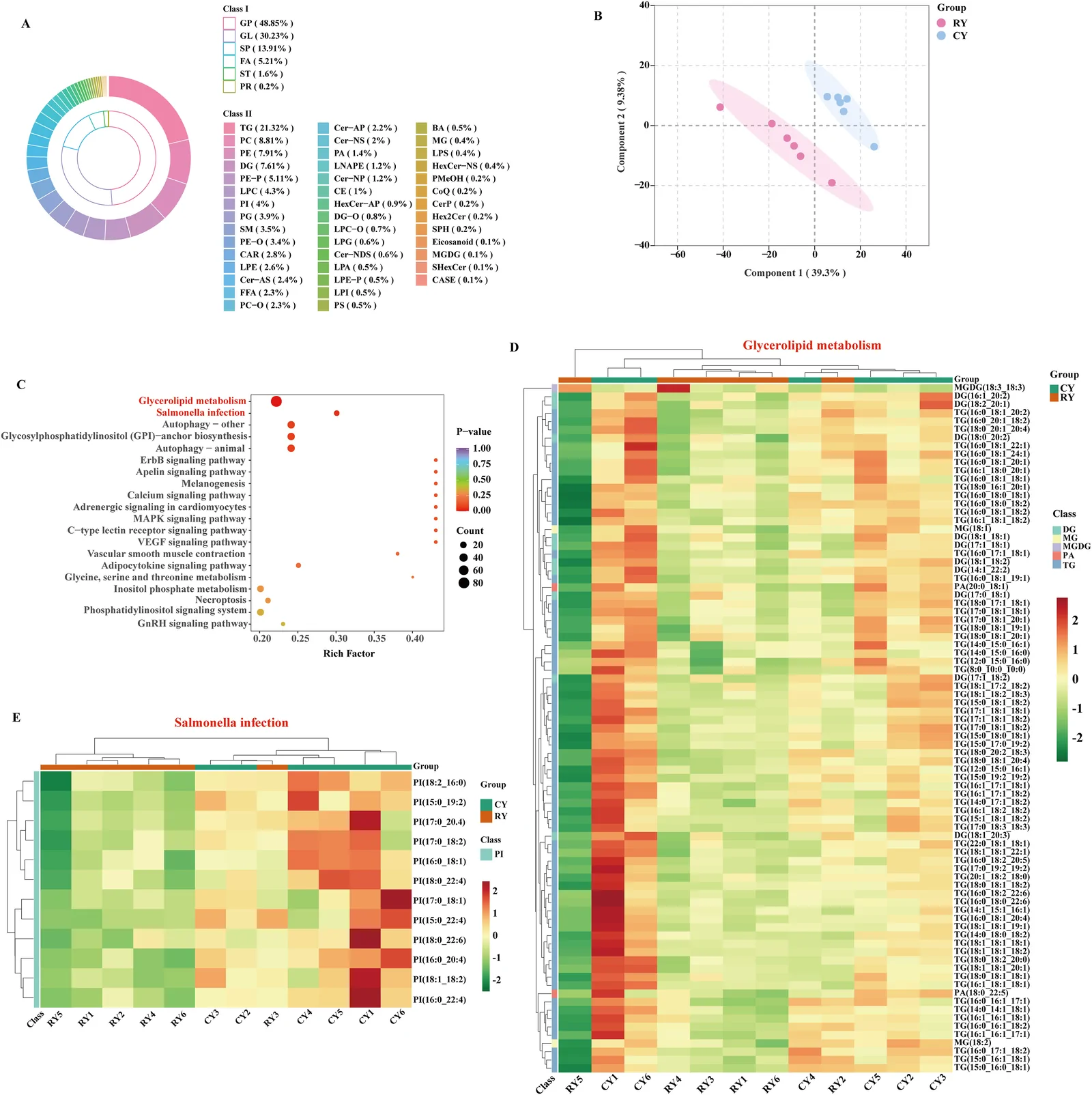
**Lipidomic profiling of raw (RY) and cooked (CY) quail egg yolks.** (A) Classification and relative abundance of identified lipid species. (B) PLS-DA score plot showing separation of lipid profiles between RY and CY samples. (C) KEGG pathway enrichment analysis based on differential lipid species. (D) Heatmap showing differential glycerolipid species mapped to the glycerolipid metabolism KEGG annotation. (E) Heatmap showing differential phospholipid species mapped to the Salmonella infection KEGG annotation.

To characterize lipid remodeling induced by thermal processing and its potential association with aroma changes, comprehensive lipidomic profiling was performed on raw and cooked quail egg yolks. A total of 1058 lipid species were identified, belonging to six major classes, including glycerophospholipids (GP, 48.85%), glycerolipids (GL, 30.23%), sphingolipids (SP, 13.91%), fatty acids (FA, 5.21%), sterol lipids (ST, 1.60%), and prenol lipids (PR, 0.20%) (Fig. 3A). Among these, triacylglycerols (TGs), phosphatidylcholines (PCs), phosphatidylethanolamines (PEs), diglycerides (DGs), and monoglycerides (MGs) were the predominant subclasses, indicating that neutral lipids and membrane phospholipids constitute the major components of the quail yolk lipidome (Luo et al., 2023). TGs and DGs serve as important reservoirs of unsaturated fatty acyl chains and are susceptible to thermal oxidation and hydrolysis (Zhai et al., 2025), suggesting their potential contribution to the formation of lipid-derived volatiles, particularly aldehydes and ketones. In addition, glycerophospholipids such as PCs and PEs are major membrane phospholipids that frequently contain unsaturated fatty acyl chains and are susceptible to oxidative degradation during heating (Liu et al., 2025). Alterations in these phospholipid species may be associated with lipid oxidation during heating and therefore may contribute to aroma-related chemical transformations (Lee et al., 2025).

PLS-DA showed clear separation between raw (RY) and cooked yolk (CY) samples (Fig. 3B), indicating that thermal processing substantially altered the yolk lipid profile. A total of 185 lipid species were significantly changed after heating. The differential lipids (VIP > 1, *P* < 0.05) are summarized in Table S5. KEGG pathway enrichment analysis showed that differential lipid species were enriched in annotations related to glycerolipid metabolism and membrane lipid processes (Fig. 3C), suggesting that differential lipids were mainly associated with glycerolipid metabolism and membrane lipid-related pathways. Hierarchical clustering revealed distinct distribution patterns of differential glycerolipids, including TGs, DGs, MGs, phosphatidic acids (PA), and phosphatidylinositols (PI), between RY and CY samples (Fig. 3D). Several TG and DG species were increased in cooked yolks, suggesting enhanced lipid transformation during heating. In addition, phospholipid subclasses such as PCs and PEs exhibited significant alterations (Fig. 3E), reflecting heat-induced changes in membrane lipid composition. Overall, these results indicate that thermal processing modifies both storage lipids and membrane phospholipids, altering the abundance of oxidation-prone lipid species, particularly TGs and DGs. These coordinated changes in storage lipids, membrane phospholipids, and volatile compounds suggest a close association between lipid remodeling and aroma evolution during thermal processing. However, because the present lipidomics dataset primarily characterized lipid molecular species rather than complete fatty acyl compositions, the specific fatty acid precursors responsible for individual volatile oxidation products could not be unequivocally assigned based on the present dataset. Future studies integrating targeted fatty acyl profiling, fatty acyl-resolved lipidomics, and biochemical validation will be valuable for establishing direct precursor–product relationships.

### 3.4 Remodeling of water-soluble metabolites during thermal processing

**Fig. 4:**
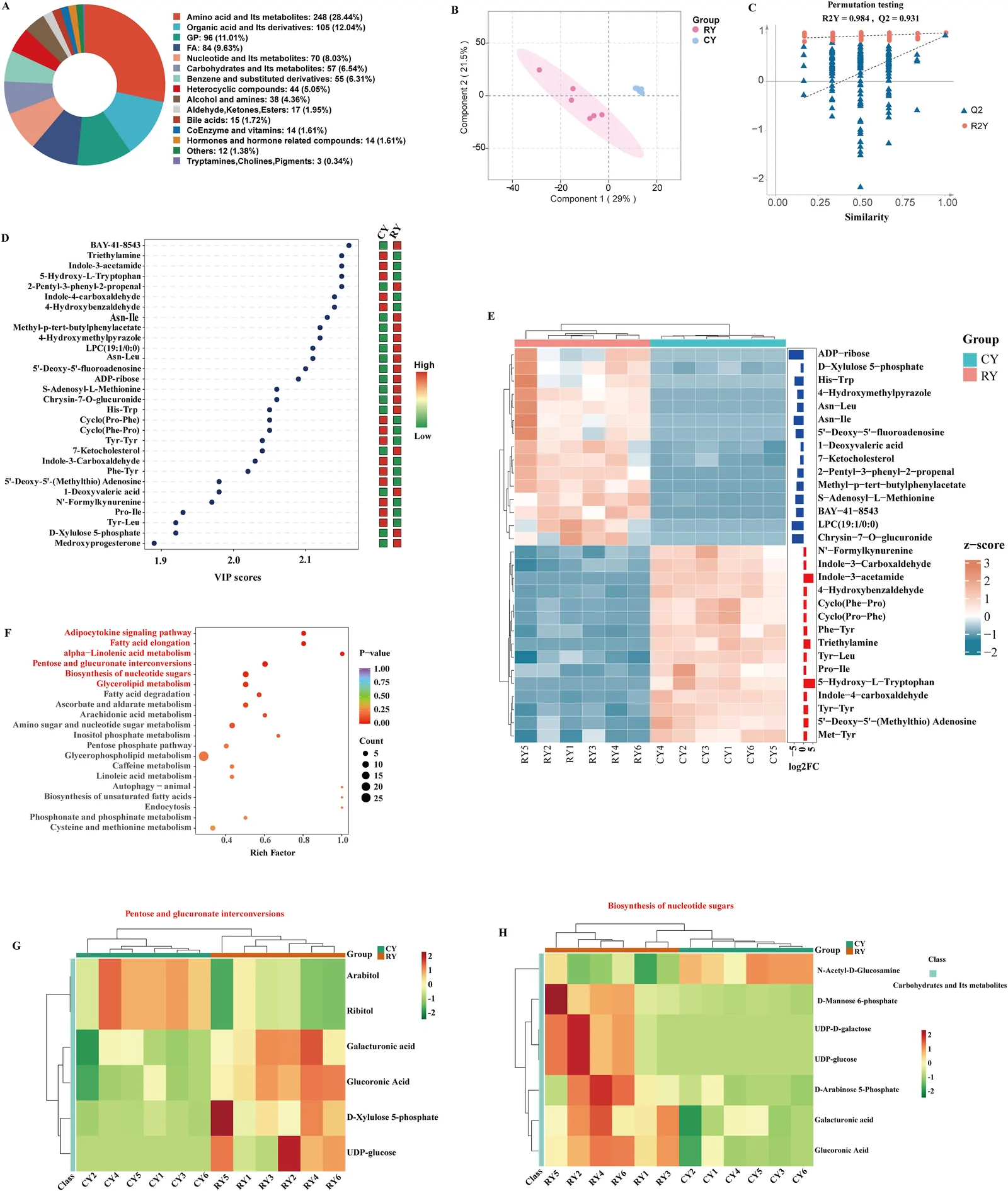
**Metabolomic characterization of raw (RY) and cooked (CY) quail egg yolks.** (A) Classification and number of identified metabolites. (B) PLS-DA score plot showing separation between RY and CY samples. (C) Permutation test validating the PLS-DA model. (D) Variable importance in projection (VIP) score plot of differential metabolites between the raw yolk (RY) and cooked yolk (CY) groups. Metabolites are ranked according to their VIP scores, and the colored squares indicate their relative abundance in each group. (E) Heatmap showing the relative abundance of selected differential metabolites. (F) KEGG pathway enrichment analysis based on differential metabolites. (G) Heatmap of metabolites annotated to pentose and glucuronate interconversions. (H) Heatmap of metabolites annotated to fatty acid metabolism.

To characterize metabolic changes associated with heat-induced flavor development, widely targeted metabolomics was performed on raw (RY) and cooked (CY) quail egg yolks. A total of 872 metabolites were identified (Fig. 4A), covering diverse chemical classes. Amino acids and their derivatives represented the largest proportion (248 metabolites, 28.44%), followed by organic acids and their derivatives (105 metabolites, 12.04%) and glycerophospholipids (9.01%), with additional contributions from fatty acids, nucleotides, carbohydrates, and other minor metabolites. The abundance of amino acid- and lipid-related metabolites suggests that these metabolite classes may represent important metabolite pools associated with flavor development during thermal processing (Luo et al., 2023). PLS-DA showed clear separation between RY and CY samples (Fig. 4B), indicating pronounced alterations in metabolite profiles after heating. Permutation testing further confirmed the reliability of the model, with a Q^2^ intercept of approximately −0.841 (Fig. 4C), suggesting good predictive performance without overfitting. These results demonstrate that thermal processing significantly alters the metabolic composition of quail egg yolk. VIP score analysis further identified the metabolites contributing most strongly to the discrimination between the RY and CY groups (Fig. 4D). The relative abundance patterns of representative differential metabolites are presented in Fig. 4E, where hierarchical clustering clearly separated the RY and CY groups, further supporting the extensive metabolic alterations induced by thermal processing.

To further characterize the functional annotations associated with these differential metabolites, 180 significantly altered metabolites (VIP > 1, *P* < 0.05) were subjected to KEGG pathway enrichment analysis (Fig. 4F). The complete list of differential metabolites associated with flavor formation (VIP > 1, *P* < 0.05) is provided in Table S6. The significantly altered metabolites were primarily enriched in KEGG annotations related to pentose and glucuronate interconversions, fatty acid metabolism, and α-linolenic acid metabolism, indicating that carbohydrate- and lipid-related metabolites were prominently represented among the differential metabolites (Fig. 4F–H). Metabolites annotated to the pentose and glucuronate interconversion, including UDP-glucose and D-xylulose-5-phosphate, showed significant alterations (Fig. 4G), indicating changes in nucleotide sugar- and carbohydrate-related metabolites. Given the established involvement of reducing sugars in Maillard reactions during thermal processing, these changes may be associated with the chemical environment relevant to Maillard-related aroma development (Li et al., 2025; Luo et al., 2023; Zhou et al., 2024a, Zhou et al., 2024b). Changes in several fatty acid-related metabolites were observed after heating, suggesting alterations in lipid-derived metabolites that may be associated with lipid oxidation during thermal processing (Li et al., 2025; Luo et al., 2023). Together with the extensive alterations observed in glycerophospholipids and glycerolipids in the lipidomic analysis, these findings suggest coordinated remodeling of lipid composition during thermal processing, while the metabolomic results provide functional annotation of the differential metabolites rather than evidence of pathway-level activity. Metabolites annotated to α-linolenic acid metabolism also showed significant alterations during thermal processing, indicating changes in polyunsaturated fatty acid-related metabolites (Wen et al., 2019). These metabolite changes may be associated with the observed alterations in volatile profiles, although direct precursor–product relationships cannot be established from the present data. Previous studies have shown that metabolite responses to thermal processing vary depending on food matrices and processing conditions (Chen et al., 2026; Li et al., 2026). Consistent with these observations, the present study identified coordinated alterations in sugar- and lipid-related metabolites, suggesting that these metabolic changes may be associated with aroma development during thermal processing. Collectively, these coordinated metabolic alterations suggest changes in metabolite pools associated with lipid oxidation and Maillard-related reactions during thermal processing, which may contribute to the observed changes in aroma profiles.

### 3.5 Integrated correlation analysis reveals associations of differential lipids and metabolites with aroma-related compounds

To investigate associations among aroma-related VOCs, differential lipid species, and metabolites, Pearson correlation analysis was performed. Because the analysis compared only raw and cooked yolk samples, the observed correlations were interpreted as statistical associations rather than direct biochemical relationships. Distinct correlation patterns were observed among representative aroma compounds, suggesting different metabolic associations during thermal processing. Correlation analysis between the top 15 GC–IMS-derived VOCs (VIP > 1) and OAV values, presented in Fig. S1B , revealed clear clustering patterns related to processing conditions. VOCs enriched in cooked yolk (CY), including propanal-M, 3-penten-2-one, 2-butanone, dimethyl sulfide, 2-methyl-2-propanol, (E)-2-pentenal, 3-methyl-2-butenal, and 1-penten-3-ol, showed significant positive correlations with benzeneacetaldehyde (floral, honey-like), whereas they were negatively correlated with decanal (fatty, citrus-like), which was more abundant in raw yolk (RY). This opposite correlation pattern suggests coordinated shifts in volatile composition during heating, with changes in the relative abundance of volatile compounds associated in previous studies with lipid- and amino acid-related reactions. The negative association between CY-enriched VOCs and decanal may reflect differences in lipid-related volatile profiles during cooking, whereas the positive association with benzeneacetaldehyde is consistent with previous reports linking this compound to thermal reactions involving amino acids and reducing sugars.

Decanal showed significant positive correlations (r > 0.4, *P* < 0.05) with multiple glycerolipid species, particularly triacylglycerols (TGs) and cholesterol-associated lipids (TC). The complete correlation coefficients are provided in Table S7. Previous studies have shown that glycerolipids containing unsaturated fatty acids (e.g., C18:1 and C18:2) may contribute to the formation of aldehydes through lipid oxidation during thermal processing (H. Li et al., 2025). Accordingly, the observed positive correlations between decanal and several glycerolipid species are consistent with this possibility. However, the present correlation analysis supports only associations between individual lipid species and volatile compounds and does not establish direct precursor–product relationships. In contrast, benzeneacetaldehyde exhibited predominantly negative correlations with TG and TC species, suggesting that its accumulation was associated with metabolic changes different from those related to lipid-derived aldehydes. Integration with metabolomic data further revealed that benzeneacetaldehyde was negatively correlated with arabitol and ribitol, two metabolites associated with pentose metabolism and reducing sugar transformation. These coordinated associations are consistent with previous reports suggesting that amino acids and reducing sugars may participate in Maillard-related reactions during heating, although the present data do not directly demonstrate these reaction pathways.

**Fig. 5:**
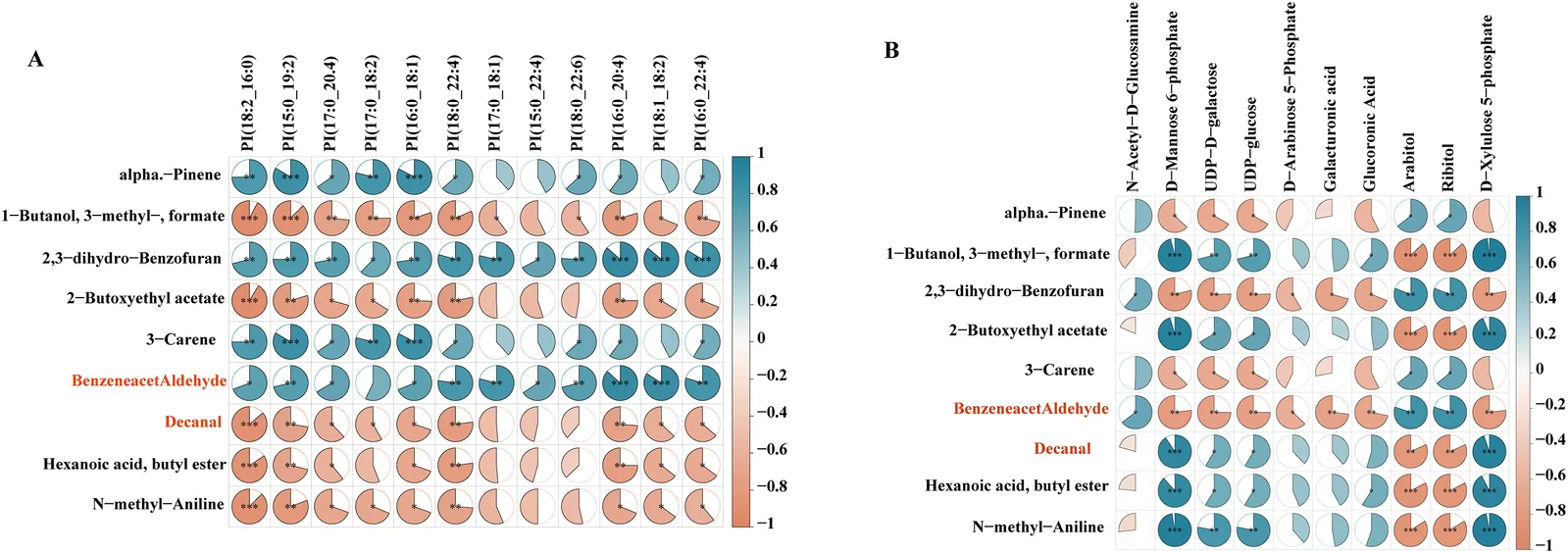
**Correlation analysis between differential lipids/metabolites and aroma-related compounds in quail egg yolk.** (A) Pearson correlation analysis between significantly differential lipid species and differential volatile compounds (OAV > 1, VIP > 1, *P* < 0.05). (B) Pearson correlation analysis between significantly differential metabolites and differential volatile compounds (OAV > 1, VIP > 1, *P* < 0.05). Significance levels are indicated as *P < 0.05, **P < 0.01, and ***P < 0.001.

Compared with benzeneacetaldehyde, decanal showed positive correlations with arabitol and ribitol, suggesting that alterations in carbohydrate-related metabolites may be associated with changes in lipid-derived volatile profiles during heating (Fig. 5A-B). These findings suggest coordinated changes between carbohydrate-related metabolites and lipid-derived aroma compounds during thermal processing. Collectively, the integrated multi-omics analysis identified two distinct patterns of metabolite–volatile associations: one characterized by glycerolipid-associated correlations with fatty aldehydes, represented by decanal, and the other characterized by amino acid- and sugar-related correlations with aromatic aldehydes, represented by benzeneacetaldehyde (Tang et al., 2025). These association patterns provide an integrated view of metabolic changes accompanying thermal processing rather than direct evidence for independent aroma-formation pathways. Overall, the present integrated multi-omics analyses suggest that thermal processing is accompanied by coordinated remodeling of lipid- and amino acid-related metabolites together with changes in volatile composition, which may collectively contribute to the characteristic aroma profile of cooked quail egg yolk. Compared with previous studies on egg flavor development during thermal processing that primarily focused on volatile identification or individual lipid oxidation products, the present study integrates volatilomics, lipidomics, and metabolomics to reveal coordinated association patterns among lipid remodeling, metabolite changes, and aroma compounds. These findings provide a systems-level framework for understanding aroma formation during thermal processing. Nevertheless, the present integrated multi-omics analyses identify coordinated associations rather than direct biochemical relationships, and further biochemical validation will be required to determine causal links between glycerophospholipid remodeling and aroma volatile formation.

## 4 Conclusion

This study integrated volatile profiling, lipidomics, and widely targeted metabolomics to characterize the metabolic changes associated with aroma development in quail egg yolk during thermal processing. Benzeneacetaldehyde and decanal were identified as representative volatile compounds and showed distinct associations with lipid- and amino acid-related metabolites, suggesting coordinated changes in volatile composition and metabolite profiles during heating. Integrated metabolomic analysis further indicated that differential metabolites were mainly enriched in KEGG annotations related to fatty acid metabolism, pentose and glucuronate interconversions, and α-linolenic acid metabolism, indicating that lipid- and carbohydrate-related metabolites were prominently represented among the differential metabolites. Overall, this study provides an integrated multi-omics perspective on the coordinated changes in volatile compounds, lipids, and metabolites accompanying thermal processing, contributing to a better understanding of aroma development in cooked quail egg yolk. However, because only a single standardized boiling condition was investigated, the findings of the present study are limited to this specific thermal treatment and should not be generalized to other thermal processing conditions. Future studies incorporating different heating temperatures and durations, together with targeted biochemical validation, are warranted to further elucidate the dynamic relationships between processing intensity, lipid remodeling, and aroma formation, thereby supporting the development of flavor-oriented processing strategies.

## Authorship contribution statement

Cui Ma:Writing - Original Draft, Validation, Formal analysis, Investigation, Data curation. Xiaoyue Yu: Investigation, Formal analysis. Jinsong Pi: Validation, Visualization. Hao Zhang: Visualization. Yuejin Pu: Investigation. Wentao Ke: Investigation. Yan Wu: Writing Review & Editing, Funding acquisition, Resources, Supervision, Project administration.

## Declaration of competing interest

The authors have no relevant financial or non-financial interests to disclose.

## Figures and Tables

**figs1:**