# Supplementary material for: Lipid remodeling is associated with aroma formation during thermal processing of quail egg yolk: an integrated multi-omics study
Source: Curr Res Food Sci. 2026 Sep 12;13:101559. doi: 10.1016/j.crfs.2026.101559 (PMC13601042; doi:10.1016/j.crfs.2026.101559)
Supplement: Supplementary file 1 [file mmc1.docx]

**Supplementary Figure legend**

**Figure S1 Pearson correlation analysis of representative volatile organic compounds (VOCs) identified in quail egg yolk.** (A) Pairwise Pearson correlation analysis among representative VOCs identified by GC–IMS. (B) Pearson correlation analysis between VOCs identified by GC–IMS and HS-SPME–GC–MS, showing the relationships between volatile compounds detected by the two analytical platforms.
